# Exploring the correlation between cognitive impairment and serum troponin I in the Qinghai Plateau region

**DOI:** 10.1002/alz70856_101855

**Published:** 2025-12-25

**Authors:** Changbiao Chai, Aiqin Zhu

**Affiliations:** ^1^ Department of Geriatrics, Qinghai People's Hospital, Xining, China; ^2^ Graduate School of Qinghai University, xining, China; ^3^ Qinghai Provincial Hospital, Qinghai University, Xining, China; ^4^ Clinical Research Center for Geriatric Diseases of Qinghai, Xining, China

## Abstract

**Background:**

Research has found that high levels of serum myocardial necrosis markers, specifically Troponin I (TnI), can affect heart pumping, leading to an increased risk of vascular disease and cognitive impairment. TnI can also be affected by hypoxia and exerrese. Our study investigates the correlation between TnI levels and multiple cognitive domains in patients with mild cognitive impairment(MCI) and Alzheimer's disease(AD) in hypoxic environment.

**Method:**

Using a cross‐sectional approach, we selected 535 middle‐aged and elderly individuals(age 64.18 ±10.42 years) from the Qinghai region (average altitude < 3,000 meters). Participants were categorized into three groups based on their Montreal Cognitive Assessment (MoCA) scores: 318 with normal cognition, 135 with MCI, and 82 with AD. Collect demographic and general clinical data, assess with multidimensional cognitive scales, and measure blood biomarkers such as serum troponin I (TnI) levels.

**Result:**

There were statistically significant differences among the three groups in terms of age, gender, education level, occupation, height, weight, smoking, drinking, marital status, and living conditions (*p* <0.05). Compared with the cognitively normal group, the MCI group showed significantly lower Pulse Oximetry Saturation (SpO_2_) levers (*p* <0.05); serum TnI values increased in both the MCI and AD groups (*p* <0.05, *p* <0.01). The ordered logistic regression results indicate that weight (*p* = 0.012), instrumental activities of daily living ability (IADL) (*p* = 0.004), albumin (ALB) (*p* = 0.001), C‐reactive protein (*p* = 0.033), and TNI (*p* = 0.026) were risk factors for the MCI and AD. Male (*p* = 0.006), stop drinking (*p* = 0.013) and good basic activities of daily living (BADL) (*p* = 0.031) are protective factors. Correlation analysis showed that SpO₂ was positively correlated with Symbol‐Digit Modalities Test (SDMT) (r_s_=0.103), Verbal Fluency Test (VFT) (r_s_=0.111), and negatively correlated with Digit Span Test (DST) (r_s_=‐0.125), IADL (r_s_=‐0.185), and Geriatric Depression Scale (GDS) (r_s_=‐0.125) (*p* <0.05). TnI is positively correlated with Stroop color‐word test (Stroop‐C) (r_s_=0.208) and IADL (r_s_=0.185) (*p* = 0.01), while it is negatively correlated with the Auditory Verbal Learning Test ‐ Delayed Recall (AVLT) (r_s_=‐0.240), SDMT (r_s_=‐0.256), and VFT (r_s_=‐0.191) (*p* <0.01).

**Conclusion:**

Elevated TnI is a risk factor for MCI and AD patients in hypoxic environments. There is a correlation between TnI and SpO₂ levels were associated with memory, executive function, attention, and language skills.

